# Polymorphisms of pharmacogenetic candidate genes affect etomidate anesthesia susceptibility

**DOI:** 10.3389/fgene.2022.999132

**Published:** 2022-09-28

**Authors:** Lulin Ma, Yan Huang, Shiqian Huang, Feng Xu, Yafeng Wang, Shuai Zhao, Daling Deng, Yuanyuan Ding, Tianhao Zhang, Wenjing Zhao, Xiangdong Chen

**Affiliations:** ^1^ Department of Anesthesiology, Union Hospital, Tongji Medical College, Huazhong University of Science and Technology, Wuhan, China; ^2^ Department of Anesthesiology, The First People's Hospital of Jiangxia District, Wuhan, China; ^3^ Department of Anesthesiology, Zhongnan Hospital of Wuhan University, Wuhan, China

**Keywords:** single nucleotide polymorphisms, etomidate, susceptibility, GABRA2, GABRB2, CYP2C9, UGT1A9

## Abstract

**Purpose:** Etomidate is widely used in general anesthesia and sedation, and significant individual differences are observed during anesthesia induction. This study aimed to explore the molecular mechanisms of different etomidate susceptibility at the genetic level.

**Methods:** 128 patients were enrolled in the study. The bispectral index (BIS), mean arterial pressure (MAP) and heart rate (HR) were recorded when the patients entered the operating room for 5 min, before the administration of etomidate, 30 s, 60 s, 90 s, 120 s and 150 s after the administration of etomidate, and the corresponding single nucleotide polymorphisms (SNPs) were analyzed.

**Results:** Significant individual differences were observed in etomidate anesthesia. The results of two-way ANOVA showed that CYP2C9 rs1559, GABRB2 rs2561, GABRA2 rs279858, GABRA2 rs279863 were associated with the BIS value during etomidate anesthesia; UGT1A9 rs11692021 was associated with the Extended Observer’s Assessment of Alertness and Sedation (EOAA/S) score during etomidate anesthesia; GABRB2 rs2561 was associated with MAP. Multiple linear stepwise regression model results showed that CYP2C9 rs1559, GABRA2 rs279858 and GABRB2 rs2561 were associated with the BIS value and UGT1A9 rs11692021 was associated with the EOAA/S score; GABRB2 rs2561 was associated with MAP.

**Conclusion:** GABRA2 rs279858, GABRB2 rs2561, CYP2C9 rs1559 and UGT1A9 rs11692021 are the SNPs with individual differences during etomidate anesthesia. This is the first to study the SNPs of etomidate, which can provide certain evidence for the future use of etomidate anesthesia and theoretical basis for precision anesthesia.

## Introduction

Etomidate is widely used in general anesthesia and sedation, especially in patients with cardiovascular disease, because it does not inhibit sympathetic tone or myocardial function (Morgan et al., 1975) and has minimal changes in blood pressure and heart rate in patients at typical anesthesia induction doses (Scorgie, 1983). Animal experiments have shown that etomidate is associated with minimal hemodynamic changes or respiratory depression (Forman, 2011). In addition, clinical studies have also shown that etomidate has little effect on cardiovascular disease in patient (Wagner et al., 2014; Chung et al., 2020). However, intravenous etomidate also has corresponding side effects. Involuntary muscle movements often occur with large doses of etomidate (Doenicke et al., 1999; Nyman et al., 2011). Adrenal cortical function can be suppressed by previous or high-dose etomidate use (Ge et al., 2013). Some studies have found that adrenal function can be suppressed after a single bolus injection and can last for 48 h in critically ill patients, but whether it has clinical significance is still controversial (den Brinker et al., 2008; Tekwani et al., 2010). Given the advantages and side effects of etomidate, precise dosing is particularly important to ensure the safety and efficacy of anesthesia, which is a key indicator for assessing the quality of anesthesia (Haller et al., 2009).

It is found that different patients have different sensitivity to etomidate. Keita et al. found that factors such as age could change the sensitivity of patients to etomidate (Keita et al., 1998). However, studies at the genetic level have not yet been reported in the literature. Genetic variability represents a fundamental source of interindividual variability in drug dosage (Amstutz and Carleton, 2011), with single nucleotide polymorphisms (SNPs) being critical in determining patient’s response to drugs (Roden et al., 2019). A previous study of our group found that differences at the genetic level could significantly affect the sensitivity of patients to propofol (Zhong et al., 2017). We therefore hypothesized that the diversity of SNPs could affect the susceptibility of different patients to etomidate.

Gamma-aminobutyric acid type A (GABAA) receptors have been confirmed to be the target of etomidate (Franks, 2006). Etomidate mainly acts on the α and β subunits of GABAA (Forman, 2011). Besides, Etomidate inhibits the adrenal cortical axis by inhibiting the enzyme 11β-hydroxylase and mainly is metabolized by hepatic esterase (Valk and Struys, 2021). Etomidate metabolism in animals and humans depends on hepatic esterase activity, which hydrolyzes it to a carboxylic acid and an ethanol leaving group (Forman, 2011). Activation of α2-adrenergic receptors mediates the cardiovascular effects (Paris et al., 2003) and sedative effects of etomidate (Paris et al., 2007). Paris et al. found that etomidate acted as an agonist of α2 adrenergic receptors and its effect on blood pressure was mainly manifested in the increase of blood pressure *in vivo* mediated by α2B receptors (Paris et al., 2003); They also demonstrated that etomidate could interact with α2-adrenoceptors and lack of α2-adrenoceptor activity might play an important role in mediating etomidate anesthesia in mice (Paris et al., 2007). In addition, the mechanisms of etomidate itself still needs to be studied (Restrepo et al., 2009), but it is similar to propofol. Therefore the mechanisms of propofol is also considered. Combining the above action targets and metabolic mechanisms of etomidate and propofol with the related data in the national library of medicine (NIH), possible SNPs were screened out and corresponding studies were carried out to explore the effect of gene level on etomidate sensitivity.

## Materials and methods

### Patient recruitment

This study was performed in accordance with the Declaration of Helsinki and was approved by the Ethics Committee of Tongji Medical College, Huazhong University of Science and Technology (Ethics Approval Number: 2019-S1205). All patients signed informed consent. A total of 128 participants undergoing elective ear nose and throat (ENT) surgery with general anesthesia were recruited from december 2019 to november 2020 at Union Hospital, Tongji Medical College, Huazhong University of Science and Technology. All participants denied blood ties. The inclusion criteria were as follows: 1) patients undergoing elective ENT with general anesthesia; 2) age between 20-55 years old; 3) no history of surgery; 4) no history of drug use and addiction, such as opioids, opium, heroin, marijuana, Meth, ecstasy and K powder, etc; 5) no hypertension, coronary heart disease, diabetes and other medical history; 6) ASA I or II. Exclusion criteria: 1) History of allergy or drug dependence; 2) Pregnancy or breastfeeding; 3) Abnormal liver and kidney function; 4) History of hypertension, diabetes, coronary heart disease and other cardiovascular diseases; 5) Patients with any neurological or psychological disease; 6) Other diseases that may affect the test results, for example, patients with adrenal insufficiency. The preoperative visits were all conducted by the same anesthesiologist.

### Anaesthesia procedure and monitoring

The patients were not given premedication before surgery. After the patients entered the operating room, the non-invasive blood pressure, electrocardiogram, bispectral index (BIS) and blood oxygen saturation of the patient were routinely monitored, and the Extended Observer’s Assessment of Alertness and Sedation (EOAA/S), mean arterial pressure (MAP), heart rate (HR) and BIS values were recorded for 5 min after the patient entered the room (T1), before the administration of etomidate (T2), 30 s (T3), 60 s (T4), 90 s (T5), 120 s (T6) and 150 s (T7) after the etomidate administration. The regular dose of etomidate takes 15–20 s to take effect, the effect reaches its peak in about 1 min, and the duration of action is about 2–3 min (van den Heuvel et al., 2013). In addition, in clinical experiments, it was observed that the BIS value of some patients had increased at 180s after the administration of etomidate. Therefore, the cut-off time of this experimental study was 150s after the etomidate administration. Etomidate was injected intravenously at 0.1 mg/kg in about 15 s. During the entire observation process, only etomidate was given, excluding the influence of other drugs. The EOAA/S score criteria were presented in Table 1 (Kim et al., 2015).

**TABLE 1:** Extended observer's assessment of alertness and sedation (EOAA/S) score.

Score	Description	Level of sedation or anaesthesia
5	Responds readily to name spoken	Minimal
4	Lethargic response to name spoken	Moderate
3	Responds after name called loudly/repeatedly	Moderate
2	Purposeful response to mild-to-moderate shaking	Moderate
1	Responds to trapezius squeeze	Deep
0T	No response to trapezius squeeze	Light general anaesthesia
0E	No response to electrical stimulation	Deeper general anaesthesia

### DNA sample collection and DNA extraction

Four milliliter venous blood was collected from each patient 10 min prior to induction of anesthesia. Samples were stored at -80°C and subsequently extracted using the TIANamp Genomic DNA kit. The Snap shot technology system was used to determine genotypes based on detection by MALDI-TOF MS (Sequenom Inc., San Diego, CA, United States).

### Statistical analysis

All statistical analyses were performed by SPSS 27.0 software (IBM, United States). Categorical variables were described with percentages, and continuous variables were presented with mean ± standard deviation (SD). Patients were divided into two groups, homozygous for the major allele, heterozygous for the major allele and homozygous for the minor allele (Zhong et al., 2017; Zhu et al., 2022). Differences between the two groups at different time points were calculated using a two-way ANOVA test, followed by Sidak’s multiple comparisons tests. The chi-square test was used to investigate the Hardy-Weinberg equilibrium (HWE) for each SNP, and *p* < 0.05 was considered as deviation from equilibrium. To further evaluate the independent influence of these clinical factors (age, genotype, gender, and BMI), multiple stepwise linear regression analysis was applied, with *p* < 0.05 indicating statistical significance. Two-tailed *p* < 0.05 was used in the statistical analysis.

## Results

### Characteristics of participants

**FIGURE 1:**
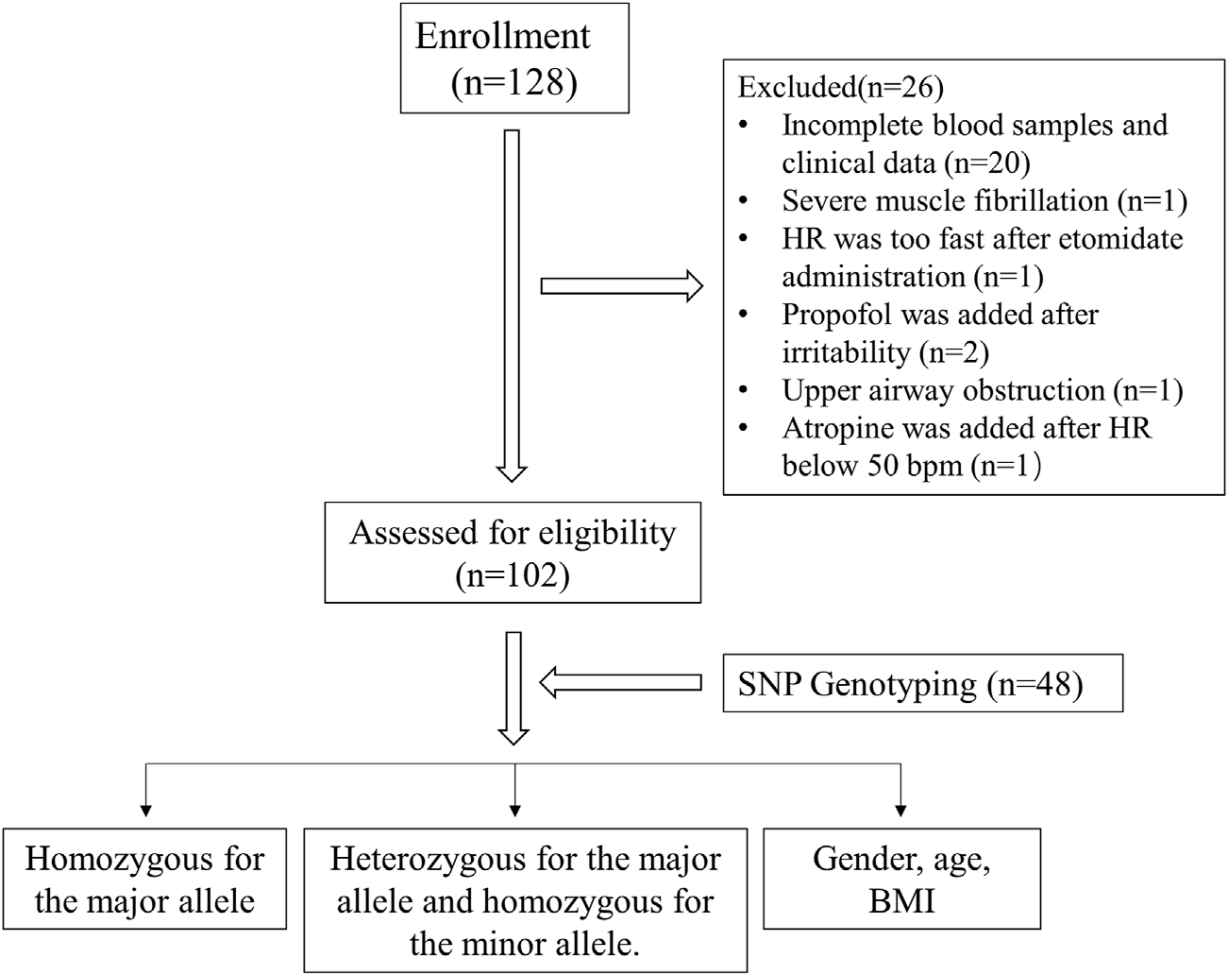
The study design and flow chart. SNP, single nucleotide polymorphism. HR, heart rate.

Ultimately, 128 patients were enrolled in the study. 26 patients were excluded due to the following reasons: 1) blood samples or clinical data were not completely recorded (*n* = 20); 2) severe muscle fibrillation led to trial termination (*n* = 1); 3) HR was too fast after etomidate administration (*n* = 1); 4) propofol was added to patients who developed irritability after etomidate administration (*n* = 2); 5) endotracheal intubation was performed immediately after the upper airway was obstructed (*n* = 1); 6) HR was less than 50 bpm after the etomidate administration, then atropine was given to increase the HR (*n* = 1). Finally, a total of 102 patients (male/female, 57/45) were collected and genotyped. The age (years) and BMI (kg/m^2^) of the participants were 37.60 ± 8.62 and 23.88 ± 3.30 respectively. The experimental flow chart was shown in Figure 1.

### Significant individual differences in bispectral index and EOAA/S scores after etomidate anesthesia

**FIGURE 2:**
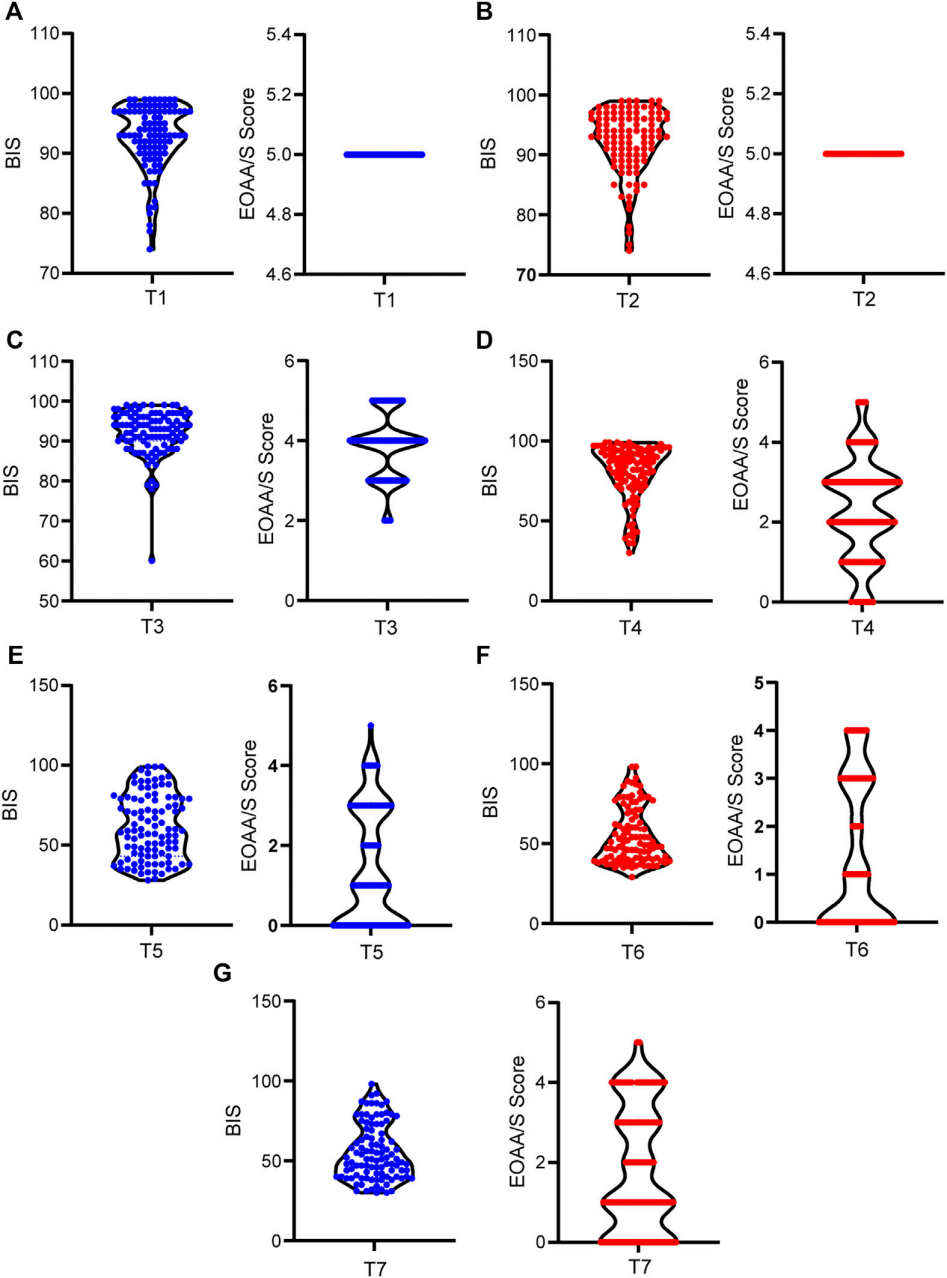
Individual differences in BIS and EOAA/S scores during etomidate anesthesia. **(A)** BIS and EOAA/S scores when the patients were quiet for 5 min after entering the operating room; **(B)** BIS and EOAA/S scores before etomidate administration; **(C)**, **(D)**, **(E)**, **(F)**, and **(G)** BIS value and EOAA/S score at 30 s, 60 s, 90 s, 120 s and 150 s. T1, 5 min after the patients entered the room; T2, before the administration of etomidate; T3, 30 s; T4, 60 s; T5, 90 s; T6, 120 s; T7, 150 s after the etomidate.

Significant individual differences were observed in etomidate induced anesthesia. Besides, there was a time-dependent decrease in BIS and EOAA/S scores. The average BIS values of patients after entering the operating room for 5 min and before etomidate administration were 92.84 ± 5.289 and 92.24 ± 5.421 respectively. All patients had an EOAA/S score of 5 at the both time points. 30 s after the etomidate, the maximum BIS of the patients was 99, and the minimum was 60. The difference between the two was 1.65 times, and the difference between EOAA/S scores was 2.5 times; The maximum value of the BIS at the 60s was 99, and the minimum was 30. The difference between the two was 3.3 times. The EOAA/S score was scattered as the highest score and the lowest score, and the difference between the two was 5 points; At 90 s, the maximum value of the BIS was 99, and the minimum value was 28. The difference between the two was 3.5 times. The difference between the EOAA/S score was 5 points. At 120 s, the maximum BIS of the patients was 98, the minimum was 29. The difference between the two was 3.4 times. The difference in the EOAA/S score was 5 points; At 150 s, the maximum BIS of the patients was 98, and the minimum was 30. The difference between the two was 3 times. The EOAA/S score differed by 5 points (Figure 2).

### Individual differences in hemodynamics after etomidate anesthesia

**FIGURE 3:**
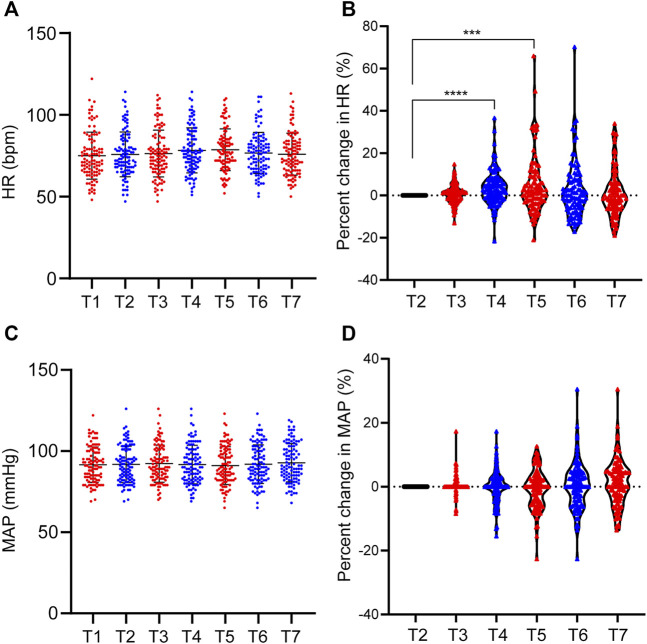
Individual differences in MAP and HR during etomidate anesthesia. **(A)** MAP distribution of patients at each time point; **(B)** MAP change rate at each time point; **(C)** HR distribution of patients at each time point; **(D)** HR change rate at each time point. T1, 5 min after the patients entered the room; T2, before the administration of etomidate; T3, 30 s; T4, 60 s; T5, 90 s; T6, 120 s; T7, 150 s after the etomidate. ***p < 0.001, ****p < 0.0001.

There were individual differences in hemodynamics during etomidate anesthesia. However, etomidate has little effect on the hemodynamics of patients. The HR was 75.06 ± 14.39 bpm when the patients were quiet for 5 min after entering the operating room, and the HR before etomidate was 75.87 ± 13.72 bpm. The HR at 30 s, 60 s, 90 s, 120 s and 150s were 76.36 ± 14.33 bpm, 78.26 ± 13.75 bpm, 78.67 ± 12.75 bpm, 76.72 ± 12.57 bpm and 75.98 ± 12.84 bpm, respectively; Compared with the basal HR, the HR change rate at 30s, 60s, 90s, 120s and 150s after etomidate anesthesia were 0.6% ± 4.4%, 3.5% ± 8.1% (*p* < 0.0001), 4.7% ± 13.4% (*p* = 0.0005), 2.1% ± 12.9% and 0.9% ± 11%, respectively; MAP was 91.84 ± 11.28 mmHg when the patients were quiet for 5 min after entering the operating room, and MAP was 75.87 ± 13.72 mmHg before etomidate anesthesia. MAP at 30 s, 60 s, 90 s, 120 s and 150s were 92.25 ± 11.40 mmHg, 91.70 ± 11.88 mmHg, 91.03 ± 11.65 mmHg, 91.96 ± 11.88 mmHg, and 92.80 ± 11.74 mmHg, respectively. Compared with the basal MAP, the MAP change rate at 30 s, 60 s, 90 s, 120 s, and 150 s were 0.5% ± 2.9%, -0.1% ± 5.0%, -0.8% ± 5.9%, 0.3% ± 7.2% and 1.2% ± 7.1%, respectively (Figure 3).

### Genotyping results

A total of 48 SNPs were detected in 102 patients. No genetic variants were found in GABRA2 rs2555, CYP11B1 rs1584, CES1 rs1066 and CYP2C19 rs1557 in all subjects, and all patients were homozygous for the major allele. GR1N2B rs3764028, ABCB1 rs1045642, BCHE rs590 and CACNA1A rs773 did not conform to Hardy–Weinberg equilibrium (HWE). The remaining 40 SNPs had genetic variation in patients and were in compliance with the HWE equilibrium (Table 2).

**TABLE 2:** Candidate gene and polymorphism list.

Gene	SNP ID	Alleles	HWE *p* value
SCN9A	rs6746030	A>C,G	0.641
CHRM2	rs1824024	C>A,G,T	0.691
5HT2A	rs6313	G>A,C	0.681
GR1N3A	rs3739722	C>T	0.075
GR1N2B	rs3764028	T>A,G	0.000
ABCB1	rs1045642	A>C,G,T	0.000
UGT1A9	rs11692021	T>C,G	0.116
GABRA2	rs279858	T>A,C	0.855
	rs567926	G>A	0.429
	rs11503014	C>G	0.528
	rs2555	C>A,G	/
	rs279827	A>C,G,T	0.645
	rs279836	T>A,G	0.523
	rs279863	C>A	0.855
GABRA1	rs490434	A>G,T	0.170
	rs2554	T>C	0.960
	rs2279020	G>A	0.831
	rs2279020	G>A	0.831
	rs2290732	A>G,T	0.831
GABRB2	rs2229944	G>A	0.060
	rs6556547	C>A,T	0.159
	rs1816071	T>A,C	0.384
	rs194072	T>A,C	0.132
	rs187269	A>G	0.094
	rs2561	A>C,G,T	0.206
	rs252944	C>G	0.132
CYP11B2	rs1585	T>A,C	0.423
HSD11B1	rs3290	G>A,C	0.367
CYP11B1	rs1584	A>T	/
PRKCB	rs9922316	T>A,G	0.960
ADORA2A	rs135	T>A,C,G	0.473
ADRA2A	rs250	- > TG	0.082
	rs1800544	G>A,C	0.702
ADRA2B	rs151	C>A,G	0.641
ADRB2	rs1042713	G>A,C	0.904
	rs154	A>C,G,T	0.389
CES1	rs1066	A>G	/
BCHE	rs590	A>C,G,T	0.000
CYP2C9	rs1057910	A>C,G	0.528
	rs1559	G>C,T	0.499
CYP2C19	rs1557	A>T	/
CYP2D6	rs1565	A>C,G,T	0.332
CYP3A4	rs1576	G>A,C	0.246
POR	rs5447	T>C	0.060
CYP1A2	rs2470890	T>C	0.498
CYP2B6	rs3745274	G>A,T	0.625
CACNA1A	rs773	T>A,G	0.039
KCNK2	rs3776	G>C	0.960

### Correlation between single nucleotide polymorphisms genotyping and etomidate-induced sedation depth sensitivity

**FIGURE 4:**
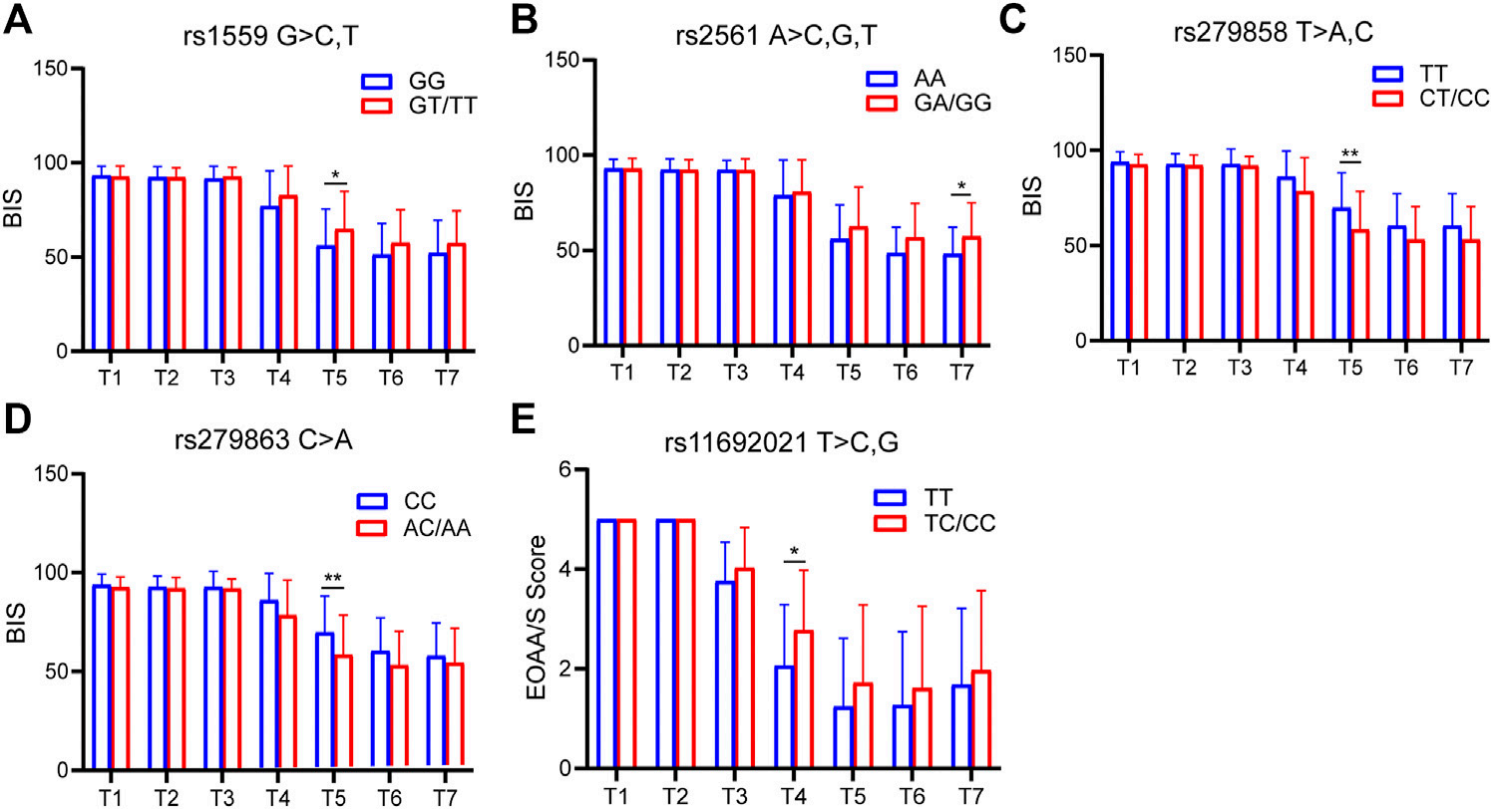
SNPs associated with BIS and EOAA/S scores. **(A)** BIS of CYP2C9 rs1559 at each time point. Compared with the homozygous for the major allele, the heterozygous for the major allele and homozygous for the minor allele had higher BIS at 90 s. The difference was statistically significant. **(B)** BIS of GABRB2 rs2561 at each time point. **(C)** BIS of GABRB2 rs279858 at each time point. **(D)** BIS of GABRB2 rs279863 at each time point. **(E)** EOAA/S score of UGT1A9 rs11692021 at each time point. T1, 5 min after the patients entered the room; T2, before the administration of etomidate; T3, 30 s; T4, 60 s; T5, 90 s; T6, 120 s; T7, 150 s after the etomidate. Two-way ANOVA was used as statistical analysis. *p < 0.05, **p < 0.01.

The sedation depth induced by etomidate was assessed using the BIS and EOAA/S scores. According to the SNP genotyping results, the patients were divided into two groups: 1) homozygous for major allele; 2) heterozygote for major allele and homozygous for minor allele. For BIS, 4 SNPs (CYP2C9 rs1559, GABRB2 rs2561, GABRA2 rs279858, GABRA2 rs279863) showed significant differences at different time points; for EOAA/S score, only one SNP (UGT1A9 rs11692021) had a statistically significant difference (Figure 4).

### Multiple linear regression analysis of the relationship between multiple factors and bispectral index as well as EOAA/S score

In order to study the effects of multiple factors (gender, age, BMI) and corresponding SNPs on BIS, a multiple linear stepwise regression model was used to perform regression analysis on these factors. The results showed that CYP2C9 rs1559 and GABRA2 rs279858 had statistical significance at T5 (120 s) after intravenous etomidate; GABRB2 rs2561 had statistical significance at T6 (120 s); GABRB2 rs2561 had statistical significance at T7 (150 s). However, there was no significant difference in age, gender and BMI at any time pointe (Table 3).

**TABLE 3:** Multiple linear regression analysis of clinical variables related to BIS.

Independent variable	Unstandardized coefficients	SE	Standardized coefficients	t	*p*
T5					
CYP2C9 rs1559	9.097	3.85	0.224	2.363	0.02
GABRA2 rs279858	−11.755	4.482	−0.249	−2.622	0.01
Model fit: R = 0.328, R2 = 0.108, adjust R2 = 0.09, DW = 1.839, F = 5.984, *p* = 0.004					
T6					
GABRB2 rs2561	8.288	4.034	0.201	2.054	0.043
Model fit: R = 0.201, R2 = 0.04, adjust R2 = 0.031, DW = 1.867, F = 4.221, *p* = 0.043					
T7					
GABRB2 rs2561	9.048	4.02	0.22	2.251	0.027
Model fit: R = 0.22, R2 = 0.048, adjust R2 = 0.039, DW = 1.777, F = 5.065, *p* = 0.027					
Min BIS					
GABRB2 rs2561	9.470	3.959	0.233	2.392	0.019
Model fit: R = 0.233, R2 = 0.054, adjust R2 = 0.045, DW = 1.755, F = 5.723, *p* = 0.019					

DW: Durbin–Watson test. SE: standard error. BMI: body mass index. Min: minimum. T1, 5 min after the patients entered the room; T2, before the administration of etomidate; T3, 30 s; T4, 60 s; T5, 90 s; T6, 120 s; T7, 150 s after the etomidate.

In order to study the effects of multiple factors (gender, age, BMI) and the corresponding SNPs on EOAA/S scores, a multiple linear stepwise regression model was used to perform regression analysis on these factors. The results showed that gender differences were statistically significant at T3 (30 s); The UGT1A9 rs11692021 was statistically significant at T4 (60 s); Gender and BMI were statistically significant at T5 (90 s); BMI was statistically significant at T6 (120 s) and T7 (150 s) (Table 4).

**TABLE 4:** Multiple linear regression analysis of clinical variables related to EOAA/S score.

Independent variable	Unstandardized coefficients	SE	Standardized coefficients	t	*p*
T3					
Gender	0.545	0.151	0.340	3.619	<0.001
Model fit: R = 0.340 R2 = 0.116, adjust R2 = 0.107, DW = 2.011, F = 13.095, *p* < 0.001					
T4					
UGT1A9 rs11692021	0.717	0.252	0.274	2.845	0.005
Model fit: R = 0.274 R2 = 0.075, adjust R2 = 0.066, DW = 2.161, F = 8.096, *p* = 0.005					
T5					
BMI	−0.109	0.043	−0.247	−2.512	0.014
Gender	0.629	0.285	0.217	2.205	0.030
Model fit: R = 0.292 R2 = 0.085, adjust R2 = 0.067, DW = 1.993, F = 4.619, *p* = 0.012					
T6					
BMI	−0.114	0.045	−0.245	−2.524	0.013
Model fit: R = 0.245 R2 = 0.060, adjust R2 = 0.050, DW = 1.982, F = 6.369, *p* = 0.013					
T7					
BMI	−0.147	0.045	−0.314	−3.303	0.001
Model fit: R = 0.314 R2 = 0.098, adjust R2 = 0.089, DW = 1.835, F = 10.913, *p* = 0.001					

DW: Durbin–Watson test. SE: standard error. BMI: body mass index. T1, 5 min after the patients entered the room; T2, before the administration of etomidate; T3, 30 s; T4, 60 s; T5, 90 s; T6, 120 s; T7, 150 s after the etomidate.

### Correlation between single nucleotide polymorphisms genotyping and hemodynamics

**FIGURE 5:**
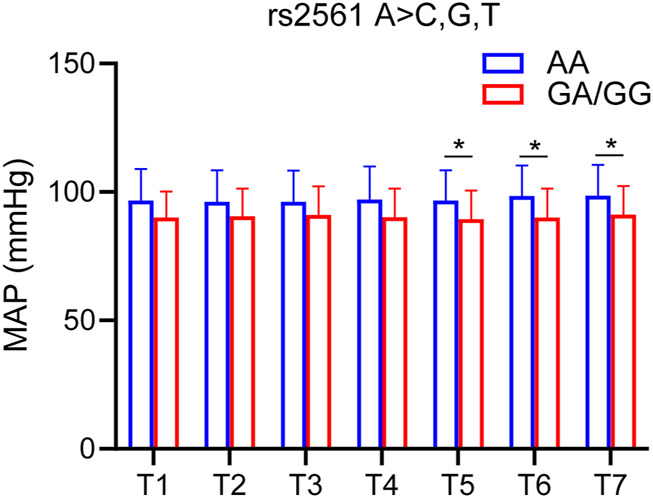
SNP associated with MAP. MAP of GABRB2 rs2561 at each time point. Compared with the homozygous for the major allele, the heterozygous for the major allele and homozygous for the minor allele had lower MAP at 90s, 120s and 150s. The difference was statistically significant. T1, 5 min after the patients entered the room; T2, before the administration of etomidate; T3, 30 s; T4, 60 s; T5, 90 s; T6, 120 s; T7, 150 s after the etomidate. Two-way ANOVA was used as statistical analysis. *p < 0.05.

MAP was used as one of the hemodynamic indexes. According to the SNP genotyping results, the patients were divided into two groups: 1) homozygous for major allele; 2) heterozygote for major allele and homozygous for minor allele. Only one SNP (GABRB2 rs2561) was significantly different in MAP (Figure 5).

## Multiple linear regression analysis of the relationship between multiple factors and MAP

In order to study the effects of multiple factors (gender, age, BMI) and the corresponding SNPs on MAP, a multiple linear stepwise regression model was used to perform regression analysis on these factors. The results showed that age and GABRB2 rs2561 were statistically significant at T3 (30s), T4 (60s) and T5 (90s); Age, GABRB2 rs2561 and BMI were statistically significant at T6 (120s) and T7 (150s) (Table 5).

**TABLE 5:** Multiple linear regression analysis of clinical variables related to MAP.

Independent variable	Unstandardized coefficients	SE	Standardized coefficients	t	*p*
T3					
Age	0.601	0.116	0.454	5.193	<0.001
GABRB2 rs2561	−5.007	2.376	−0.184	−2.107	0.038
Model fit: R = 0.492, R2 = 0.242, adjust R2 = 0.227, DW = 2.000, F = 15.801, *p* < 0.001					
T4					
Age	0.489	0.125	0.355	3.919	<0.001
GABRB2 rs2561	−6.871	2.564	−0.243	−2.680	0.009
Model fit: R = 0.432, R2 = 0.187, adjust R2 = 0.170, DW = 2.030, F = 11.365, *p* < 0.001					
T5					
Age	0.477	0.122	0.353	3.907	<0.001
rs2561	−7.173	2.506	−0.258	−2.863	0.005
Model fit: R = 0.439, R2 = 0.193, adjust R2 = 0.177, DW = 2.177, F = 11.828, *p* < 0.001					
T6					
Age	0.560	0.117	0.406	4.781	<0.001
GABRB2 rs2561	−7.482	2.421	−0.265	−3.090	0.003
BMI	0.669	0.308	0.186	2.168	0.033
Model fit: R = 0.543, R2 = 0.295, adjust R2 = 0.274, DW = 2.024, F = 13.69, *p* < 0.001					
T7					
Age	0.568	0.116	0.417	4.899	<0.001
GABRB2 rs2561	−6.540	2.398	−0.234	−2.727	0.008
BMI	0.688	0.306	0.194	2.250	0.027
Model fit: R = 0.540, R2 = 0.292, adjust R2 = 0.270, DW = 1.881, F = 13.447, *p* < 0.001					

DW: Durbin–Watson test. SE: standard error. BMI: body mass index. T1, 5 min after the patients entered the room; T2, before the administration of etomidate; T3, 30 s; T4, 60 s; T5, 90 s; T6, 120 s; T7, 150 s after the etomidate.

## Discussion

In the present study, we found that there were obvious individual differences during the etomidate anesthesia. According to the target of etomidate and its metabolic mechanisms, 48 possible SNPs were screened in this experiment. Among them, CYP2C9 rs1559, GABRA2 rs279858 and GABRB2 rs2561 were associated with BIS values during etomidate anesthesia; UGT1A9 rs11692021 was associated with MOAA/S score; In addition, GABRB2 rs2561 were associated with MAP.

### GABRA2 rs279858 and GABRB2 rs2561 were related to depth of sedation and hemodynamics

Previous studies have confirmed that etomidate mainly acts on GABAA receptors (Franks, 2006; Weir et al., 2017). A type of GABAAR closely related to anesthetics involves a combination of two alpha, two beta and gamma (α1β2γ2) (Nutt, 2006). The α and β subunit are the main acting subunit of etomidate (Forman, 2011). However several studies have found that the hypnotic effects of etomidate involve GABAARs containing β2 and β3 (Hill-Venning et al., 1997; Weir et al., 2017). GABAARs containing β1 are much less sensitive to the effects of etomidate. The affinity of etomidate is also enhanced by the presence of the γ subunit, while the α subtype has a weaker affinity (Brohan and Goudra, 2017). Therefore, the GABRB2 and GABRA2, as the main targets of etomidate, play an important role in etomidate anesthesia.

To date, there is no literature on the correlation between etomidate sensitivity and SNPs. However, some studies have reported the relationship between propofol and SNPs, while propofol and etomidate have similar mechanisms of action. Zeng et al. found that GABRB2 rs3816596 and GABRA1 rs4263535 polymorphisms were associated with susceptibility to propofol sedation. ABRA1 rs1157122 and GABRB2 rs76774144 polymorphisms were associated with the degree of blood pressure drop after propofol infusion (Zeng et al., 2022). A previous study by our group found an association between GABAA1 receptor SNP rs2279020 and sensitivity to BIS. In addition, dominant mutations in GABAA1 rs2279020 and GABAA2 rs11503014 were putatively associated with cardiovascular susceptibility to propofol anesthesia (Zhong et al., 2017). Our experimental study found that GABRB2 rs2561 was associated with the BIS value after etomidate sedation. Compared with the major gene homozygous group, the major gene heterozygous group and the minor gene homozygous group showed higher BIS values under the same conditions, but compared with the major gene homozygous group, the major gene heterozygous group and the minor gene homozygous group showed lower MAP values. Furthermore, we also found that GABRA2 rs279858 was associated with the BIS value after etomidate sedation. Compared with the major gene homozygous group, the major gene heterozygous group and the minor gene homozygous group showed lower BIS values under the same conditions. Therefore, we conclude that the GABRB2 rs2561 is related to the BIS and MAP of etomidate-induced anesthesia; GABRA2 rs279858 is related to the BIS induced by etomidate.

### CYP2C9 rs1559 and UGT1A9 rs11692021 were related to depth of anesthesia

CYP2C9 and UGT1A9 are the major metabolic genes for many drugs, including anesthetics. Propofol is primarily metabolized by CYP2B6 and CYP2C enzymes. The enzymes SULT1A1 and NQO1 are involved in the later steps of propofol metabolism (Restrepo et al., 2009). A previous study found that UGT1A9 genotype was an independent predictor of propofol concentrations immediately and 10 min after the end of continuous infusion in children. Propofol distribution constant was higher in carriers of the polymorphic UGT1A9 C allele. Carriers of the polymorphic CYP2B6 T allele received significantly lower total and initial doses of propofol (Pavlovic et al., 2020). CYP2C9 (c.1075A>C, rs1057910) was associated with BIS, target-controlled infusion (TCI)/effector concentration of propofol and TCI/plasma concentration of propofol values (Tong et al., 2021); CYP2C9*2 patients required higher propofol concentrations to achieve loss of consciousness (LOC) (Khan et al., 2014). Khan et al. found that patients with the UGT1A9-331C/T gene had higher propofol clearance than those without and required higher induction doses. UGT1A9-1818T/C patients took longer to reach the LOC (Khan et al., 2014). In addition, compared with patients carried UGT1A9 -440C/T CT and TT, those carried UGT1A9 -440C/T CC showed shorter durations of Observer’s Assessment of Alertness/Sedation (OAA/S) by up to 4 points, shorter BIS times to reach 80, and higher corresponding effect-site concentrations (Wang et al., 2017). Our results suggest that CYP2C9 rs1559 and UGT1A9 rs11692021 were closely related to the BIS and MOAA/S score induced with etomidate. Therefore, we can conclude that the metabolic pathway of etomidate is similar to that of propofol. And the genes that affect propofol metabolism also affect etomidate.

### Limitations

The sample size of this study is relatively small. We will further expand the sample size in the follow-up study to enhance the statistical power of the relevant SNP to verify the results of this study. Besides, the genes we selected are only based on the currently known polymorphisms, and many genes that have not yet been discovered due to genetic polymorphisms may have an effect on the effect of etomidate. Moreover, in this study, two-way ANOVA and multiple linear regression models were used to jointly verify the differential SNPs. However, results presented were not adjusted for multiple comparisons. When we adjusted for multiple comparisons, these associations were no longer statistically significant. The reason for this result may be related to the size of the sample. Therefore, we will increase the sample size for further verification in subsequent experiments. However, the current findings still have great inspiration, prompting us to carry out further research.

## Conclusion

In summary, our study suggested that significant individual differences were observed in etomidate induced anesthesia. GABRA2 rs279858, GABRB2 rs2561, CYP2C9 rs1559 and UGT1A9 rs11692021 are the SNPs with individual differences during etomidate anesthesia. This is the first to study the SNPs of etomidate, which can provide certain evidence for the future use of etomidate anesthesia.

## Data Availability

The original contributions presented in the study are included in the article/supplementary materials, further inquiries can be directed to the corresponding author.
